# Preclinical evaluation of the functionality of a polymer-coated sirolimuseluting stent in pigs

**DOI:** 10.1590/acb397324

**Published:** 2024-09-09

**Authors:** Sílvio César Perini, Jeanne Louise Fernandes Jesus, Alessandro Batista Soares, Rosane Angélica Ligabue, Luiz Carlos Bodanese

**Affiliations:** 1 Pontifícia Universidade Católica do Rio Grande do Sul – School of Medicine – Porto Alegre (RS) – Brazil.; 2 Pontifícia Universidade Católica do Rio Grande do Sul – Polytechnic School – Porto Alegre (RS) – Brazil.

**Keywords:** Polyurethanes, Carotid Arteries, Sirolimus, Microscopy, Electron, Scanning

## Abstract

**Purpose:**

To compare the endothelial coverage of different stents in porcine carotid arteries. Research problem: How effective are polyurethane stents (PU) and PU + rapamycin (PU + RAPA) compared to bare-metal stents on endothelial coverage by neointima in pigs after 28 days?

**Methods:**

The methodology had two phases for an interventional, experimental, prospective study, with three Moura pigs, 12 weeks old and weighing between 19 and 22.5 kg. In phase I, eight stents were implanted in carotid arteries; three stents coated with PU, three coated with PU + RAPA, and two without coating. After 28 days, phase II was carried out, consisting of euthanasia, removal of the stents, to evaluate the exposed area of the stent struts, and the percentage of endothelialization through optical microscopy and scanning electron microscopy.

**Results:**

The eight stents implanted with ultrasound sizing and post-dilation with a larger diameter balloon were analyzed by Doppler ultrasound, intravascular ultrasound, and angiography after 28 days.

**Conclusions:**

This study showed complete endothelial coverage by the endoluminal neointima of the stent struts, good integration and coverage with the arterial wall, with no exposed struts showing the presence of intimal hyperplasia (whitish tissue).

## Introduction

Arterial stents, or expandable endoprostheses, are used in the treatment of vascular diseases, such as stenosis. Peripheral artery disease (PAD) is a problem with a major impact on health. The number of affected individuals has increased worldwide from around 202 million in 2010 to 237 million in 2015^1^.

The lower limb is the most common site of PAD, affecting individuals over 25 years old, more than 20% affected individuals are over 70, and individuals are asymptomatic at first. The most common symptom is intermittent claudication that can develop into Chronic limb threatening ischemia (CLTI) with pain at rest, ulcer, and gangrene^1^^,^ 2.

Atherosclerosis is a systemic disease^3^, and PAD is associated with coronary artery disease (CAD) in 25 to 70% of cases. In 14 to 19% of cases, it is associated with carotid stenosis and in 10 to 23% of cases it is associated with renal artery stenosis^2^.

Angioplasties or by-pass are the treatments of choice for PAD with CLTI. Balloon angioplasties positively remodel the artery by compressing the plaque opening its lumen^4^. Negative remodeling, an undesirable effect, can occur by recoil due to elastic retraction of the artery wall immediately or prematurely in hours or days, by intimal hyperplasia in a few weeks to months, and by neoatherogenesis in between one or two years^5^.

The use of a bare-metal stent (BMS) keeps the artery open, preventing immediate or early negative remodeling, but it has no significant impact on the rates of late restenosis due to intimal hyperplasia and neoatherogenesis with restenosis in CAD happening in between 30 to 50% of cases in six months and restenosis in PAD happening in between 20 to 30%^4^.

Antistenotic or antiproliferative drug-eluting stents (DES) have been used since the 2000s, which reduced coronary stenosis rates to less than 5%, with structural modifications and technological advances, but we may have a rare late thrombosis rate with a significant complication, with an incidence of 0.3 to 0.6% per year, without a definitive plateau^6^. Studies on angioplasty in the femoro-popliteal territory show high restenosis rates^7^.

The use of dedicated BMS for the region showed an improvement on restenosis rates over stentless angioplasty, with a five-year patency rate of 43%^1^. The coated stents present bioabsorbable or stable polymer, which forms an interface between the metal struts and the arterial wall and can carry antiproliferative drugs with programmed release^8^.

The use of DES presents conflicting results regarding the superiority over the BMS^9^. In experimental studies, polymers may be related to stent thrombosis^10^. For example, ethylene vinyl acetate (EVA), considered a first-generation polymer, was the first polymer used in DES, resulting in a high rate of restenosis and late stent thrombosis^11^^,^ 12. These non-consensual findings in the literature demonstrate the need to develop devices, with stents modified according to the characteristics of the mesh, more biocompatible polymers, and more effective drugs^13^.

Neointimal hyperplasia is correlated with stent implantation. Although the pathophysiological mechanisms are complex, hemodynamic stress seems to represent the main etiology and occurs in response to local thrombus formation, inflammation and intimal and medial dissections promoted by the stents^14^. In humans, maximum neointimal cell proliferation occurs three to six months after stent implantation^14^.

In pigs, maximum neointimal growth for non-pharmacological stents occurs at 28 days, while for pharmacological stents, satisfactory re-endothelialization results at 28 days have been described in the literature, although incompletely, requiring further studies for an adequate comparative assessment between non-pharmacological and pharmacological stents^14^^,^ 15.

In this experimental study, the functionality of stents covered with polyurethane biopolymer (PU) and PU with rapamycin (PU+RAPA) compared BMS (control) implanted in porcine carotid arteries was evaluated. The endothelialization and neointimal formation were evaluated with optical microscopy (MO) and scanning electron microscopy (SEM).

## Methods

This is an experimental, intervention and prospective study on three female Moura pigs (numbers 66, 67, and 68) at 12 weeks of age, weighing between 19 and 22.5 kg, from the Concórdia production and consumption cooperative (Copérdia), located in Concórdia, Santa Catarina, Brazil.

The study was performed in the hemodynamics unit of the veterinary hospital of Universidade Luterana do Brasil (ULBRA), in the city of Canoas (RS), Brazil, accompanied by a veterinarian and a trained support team. The project was evaluated and approved by the Animal Use Ethics Committee of Pontifícia Universidade Católica do Rio Grande do Sul and ULBRA.

Polyurethane (PU) was synthesized from a mixture of aliphatic diisocyanates and polyols, as described in the literature^12^. This polymer is biodegradable, non-toxic, and thermal resistance. The RAPA (sirolimus 98%) was supplied by Concord Biotech. The BMS (CoCr metal struts with dimensions 3 × 15 mm, Chromium) was supplied by Bioway Biotechnologia.

**Figure 1:**
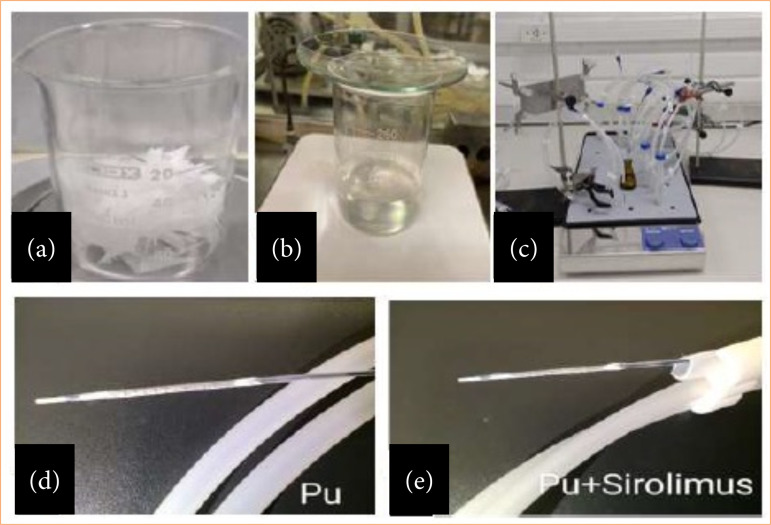
Stages of producing the polymer coating solution and forming the coating on the stent. **(a)** Polymer; **(b)** solubilization of the PU-containing solution and the PU+RAPA-containing solution; **(c)** impregnation technique on the stent; **(d)** formation of the PU-containing coating; **(e)** formation of the PU+RAPA containing coating.

The PU coating solution was prepared solubilizing PU in methyl-ethyl ketone (MEK) P.A. (Merck). The PU+RAPA coating solution was prepared solubilizing PU in MEK (Fig. 1a) and pure sirolimus/rapamycin (Concord Biotech) solubilized in ethyl acetate P.A. (Merck) (Fig. 1b).

PU: polyurethane; PU+RAPA: polyurethane with rapamycin. Source: Elaborated by the authors.

### Stent impregnation technique: PU and PU+RAPA

The impregnation of coating was made by dip-coating technique (immersion) (Fig. 1c). The BMS were immersed into PU (3% w/v) and PU+RAPA (7.4% w/v) solutions for 1 h. Afterwards, the covered stent samples were dried at room temperature for 2 h. Covered stents with a homogeneous coating with around 50–100 μm of thickness were obtained with PU and around 30–50 μm of thickness were obtained with PU+RAPA (Figs. 1d and 1e, respectively).

### Surgical and stent implant technique

**Figure 2:**
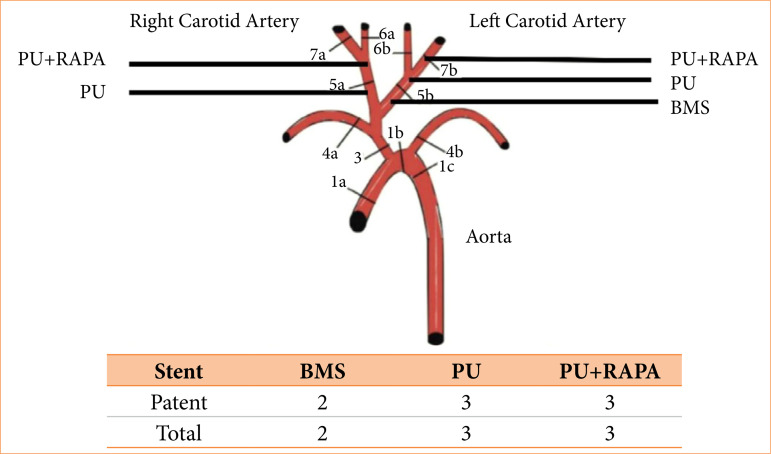
Anatomical location of the implanted stents and quantity of BMS, PU and PU+RAPA.

PU: polyurethane; PU+RAPA: polyurethane with rapamycin; BMS: bare-metal stent. Source: elaborated by the authors.

Eight stents were implanted in porcine carotid arteries, three coated with PU, three coated with PU+RAPA and two BMS, three stents in the left carotid artery and two in the right (Fig. 2)^12^. A pilot with seven stents was used to adapt the technique. The experiment was divided into two phases, phase I and phase II.

### Phase I

The animals fasted for 12 hours. They were anesthetized by a veterinarian, according to the pre-induction anesthetic protocol with 1.5 mL of azaperone (2 mg/kg), 2.5 mg of midazolan (0.5 mg/kg) and 0.4 mL of methadone (0.2 mL/kg), intramuscularly. Venous access was performed with Abocath 16 in the dorsal ear vein, and orotracheal intubation was carried out. Anesthetic induction with 0.5 mL of ketamine (1 mg/kg) and 4 mL of propofol (3 mg/kg) was performed and maintained by inhalation with 1 to 2% isoflurane (0.5 L/min) with 100% oxygen in a continuous flow.

They received prophylactic antibiotics with oxytetracycline 2 mL (1 mL/10 kg) intramuscularly during anesthetic induction, and antisepsis with alcoholic chlorhexidine and heparinization (100 IU/kg) with unfractionated heparin. Heart rate was monitored, as well as pulse oximetry. AAS 100 mg and clopidogrel 75 mg daily were initiated 48 h before phase I.

The retroperitoneal surgical approach was through a Gibson incision on the left 3 cm below the costal margin to the outer margin of the rectum abdominal sheath. The abdominal aorta was isolated. After anticoagulation, puncture was performed with an 18G needle, a 40-cm 0.035-mm guide and 10 cm 7F introducer.

Initially, the first seven carotid stents of animals 66 and 67 was implanted by angioplasty with a balloon under pressure of 8 atm (nominal), reaching 3.5 mm, to 16 atm (rupture), reaching 3.74 mm, as specified by the device under angiographic visualization only. The arteriography control image after 60 minutes showed that the right carotid stents of the first animal 66 and the bilateral carotid stents of animal 67 were occluded.

**Figure 3:**
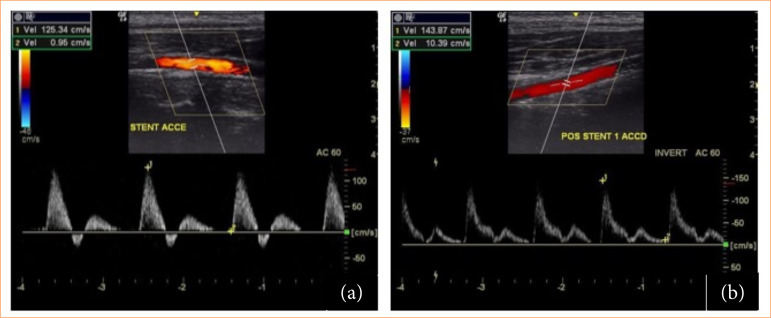
Doppler ultrasound monitoring before and after stent implantation in pigs. **(a)** Porcine doppler echography 68 pre-stent implantation; **(b)** Porcine doppler echography 68 post-implant and stent expansion.

Source: Elaborated by the authors.

**Figure 4:**
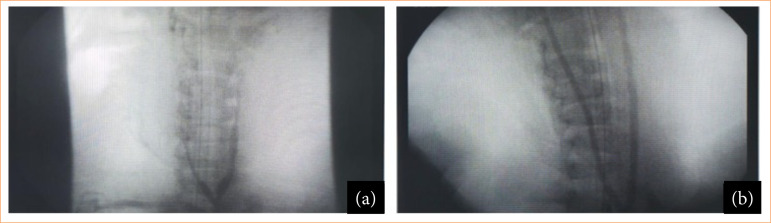
Intravascular ultrasound monitoring before and after stent implantation in pigs. **(a)** Porcine angiography 68 pre-stent implantation; **(b)** Porcine angiography 68 post-implant and stent expansion.

Source: Elaborated by the authors.

The eight stents included in the study were implanted based on carotid measurements by ultrasound (GE ultrasound) at the time of anesthetic induction and in the trans-operative period after selective catheterization of the carotid artery with a 0.014-mm guidewire and 100-cm 5F vertebral angiographic catheter and MB1 6F guide catheter under angiographic control and iodinated contrast angiography (Iopamiron 300) (Figs. 3 and 4).

The stents were implanted and expanded with a balloon at a ratio of 1:1 or 1:2 using previously estimated measurements, angiographic monitoring of the implant and opening of the device (Fig. 4). Final arteriography was performed before removing the introducer. Arteriography was carried out with prolene 6.0 and closing by planes of muscle structures, and skin was performed.

After anesthetic recovery, unlimited water was offered, and food was reintroduced after about 6 h. Analgesia was performed and monitored by the medical team for parameters of early complications or pain. After 24 h, they were taken back to the farm and remained in stalls, being fed a diet suitable for their species, and weight for 28 days.

### Phase II

The animals were anesthetized, according to the protocol. A doppler echograph was performed with flow recording and stent diameter measurements.

**Figure 5:**
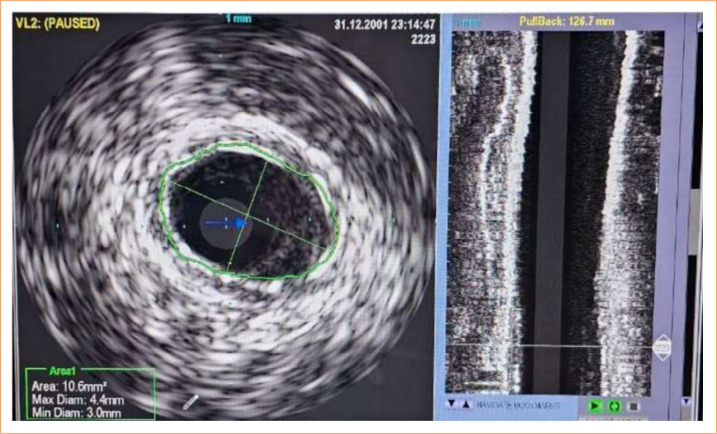
Porcine intravascular ultrasound 68 after stenting.

Source: Elaborated by the authors.

The abdominal aorta was approached through a Gibson incision on the left and direct retrograde puncture of the aorta using a 7F introducer with placement of an angiographic catheter up to the aortic arch for aortography and selective catheterization of the carotids using selective angiography and intravascular ultrasound (IVUS) (Oracle In-Vision Imaging System V 3.3.1, EndoSonics Co., United States of America), with virtual histology. Images were obtained using Eagle Eye catheters (Volcano Therapeutics Inc., United States of America) (Figs. 4 and 5).

**Figure 6:**
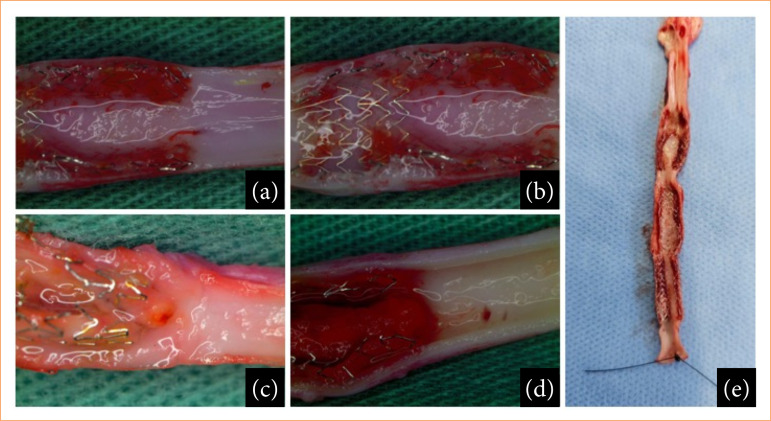
Porcine carotids with endothelization and intimal hyperplasia in optical microscopy at 100x magnification. **(a)** BMS stent; (b and c) PU stents; **(d)** PU+RAPA stent; **(e)** Surgical piece.

PU: polyurethane; PU+RAPA: polyurethane with rapamycin; BMS: bare-metal stent. Source: Elaborated by the authors.

**Figure 7:**
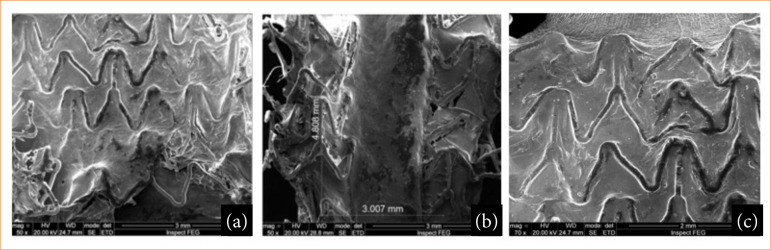
100% Endothelialization visualized by SEM in the carotids. **(a)** BMS stent; **(b)** PU stent; **(c)** PU+RAPA stent.

PU: polyurethane; PU+RAPA: polyurethane with rapamycin; BMS: bare-metal stent; SEM: scanning electron microscopy. Source: Elaborated by the authors.

After euthanasia, the stents were removed from the carotid arteries, and the anatomical pieces were opened by longitudinal arteriotomy, sectioning the stent mesh (Fig. 6). The material was placed in a 0.9% saline solution and analyzed macroscopically and using an optical microscope (Olympus SZX7 stereomicroscope). Then, it was left at a low temperature in the saline solution, impregnated and analyzed by SEM (Fig. 7).

## Results

All three animals survived until the end of the study. After the surgical approach, complications such as suture dehiscence or hernias in the aponeurotic muscle wall, bruises, infections, limb and leg ischemia, or injuries caused by the manipulation of catheters or guide wires, such as perforation and dissection, were not observed.

Seven stents were implanted, three with PU, three with PU+RAPA and one BMS to evaluate the technique. Initially, in animals 66 and 67, the choice of stent diameter measurement was based on arteriography alone. The right carotid stents from the first animal (66) and the bilateral carotid stents from animal 67 in the final control arteriography, after 60 minutes, in phase I, were occluded and excluded from the study. The eight stents that were in the left carotid artery of animal 66 and the bilateral carotid stents of animal 68 were pervious in phase II (Fig. 2).

The ultrasound and velocimetric evaluation showed that the stents were open, implanted to maintain positive remodeling, and had patency, without hemodynamic stenosis (peak systolic velocity > 2.4) on the spectral doppler (Fig. 3). In the IVUS, it was observed that the stents were pervious, without thrombosis and without stenosis. The stents were open and without fractures or structure failure, maintaining positive remodeling (Fig. 5).

Macroscopic analysis showed that the stents were well integrated into the arterial wall, without thrombosis (Fig. 6). In optical magnifying microscopy of 100x, the presence of translucent and shiny tissue characteristic of endothelialization or neointima formation was identified covering the struts of all the analyzed stents (Fig. 6). There were areas dispersed by the stents and at the transitions between the BMS, PU+RAPA and PU stents with whitish tissue characteristic of intimal hyperplasia (Figs. 1d, 1e and 6). In the SEM analysis, 100% coverage of the stent struts was observed in all the areas analyzed (Fig.7).

The SEM image in Fig. 7 can be better understood as follows: in Fig. 7a, the bare stent struts, shaped like M and W, are from the manufacturer’s industrial design. Therefore, the metallic structure was completely covered by neointimal tissue; in Fig. 7b, the cut made in the stent with PU coating shows the neointima, that is, the stent structures were covered by the neointimal tissue; in fig. 7c, the stent coating containing PU +RAPA had good neointimal tissue coverage.

For a global understanding, in order to facilitate the interpretation of the results, the terms intimal hyperplasia and neointima are described:

Intimal hyperplasia: the reconstitution of the injured vascular wall, a healing process with the formation of a thickening of the intimal layer involving the migration of smooth muscle cells from the media layer to the intima, which can generate an exaggerated response and obstruction of the vessel (artery or vein);

Neointima: scar tissue that is formed inside a blood vessel when there is injury to the intimal layer.

## Discussion

This study used pigs as study animals due to their characteristics, necessary for the proposed study: they are easy to handle and similar to humans in terms of lipid and lipoprotein metabolism, response to arterial injury with platelet aggregation and thrombus formation^13^. Atherosclerosis occurs even without a specific diet. The neointimal layer has a histology similar to that of a human being^16^^–^ 18.

Endothelial injury occurred through balloon angioplasty, as described by Schwartz et al.^19^ and by Raudales et al.^20^ and through the expansion of the stent at the time of implantation. Endothelial injury leads to intimal hyperplasia within seven to 28 days^21^^,^ 22.

The BMS, PU and PU+RAPA stents had the same cobalt chrome metal structure, with the same design and thickness of the struts, and were opened with the same balloons^23^.

The PU used is biocompatible, non-toxic and degradable (biodegradable), developed in our environment, patented and low cost. The biodegradation characteristic is important for timed release of drugs that can be controlled. The stents were impregnated using the dilution method with a 3% PU solution and 7.4% PU with rapamycin^24^.

The seven stents implanted initially were excluded, as the occlusion was corrected for technical reasons. It was observed in animals 66 and 67 that, after manipulation with the angiographic catheter and the guide wire, significant vasospasm occurred. This effect may have led to occlusion of the stents, which were undersized. The estimation of size was based solely on the initial arteriography measurement^25^.

In the eight stents in which carotid diameter measurements were taken by ultrasound and vasospasm was identified, angioplasty was performed with a 3 × 9-mm Maverick balloon under pressure until an adequate angiographic image was acquired for stent implantation.

After the 3.5-mm BMS, PU+RAPA and PU stents were implanted, the Ryujin Plus Terumo 4 × 15-mm balloon catheter was exchanged, and the stent was angioplasty and performed with a balloon compatible with the diameter of the carotid artery on ultrasound. The regions between the stents underwent angioplasty to treat and prevent negative remodeling of these regions due to vasospasm^26^.

The evaluation of the diameter of the arteries for the selection of stents is an important decision, because the animals had different weights and sizes and, consequently, arteries of different diameters.

The coating of the stents with PU did not change the opening and accommodation behavior of the stents. The behavior of the devices can be observed using the transoperative evaluation method with ultrasound in **phase I** and with ultrasound and IVUS in **phase II**. IVUS increases stent durability, since undersizing and malpositioning are causes of early thrombosis and findings of fibrin and can lead to the presence of fibrin and incomplete endothelialization in late stent thrombosis^27^.

The angiographic decision to choose the stent presented a undersizing bias, leading to distal displacement and occlusion of the stent in animal 66, weighing 22 kg, and to occlusion of the stents in animal 67 weighing 19 kg. The transoperative ultrasound examination with doppler revealed a slower, single-phase flow and distal absence, occlusion.

The artery decreased in diameter abruptly, characterizing severe vasospasm with the manipulation of the guide wire and catheter. After identifying the described changes, repeated ballooning was performed to correct the vasospasm and to adjust the stents with the reopening, employing the balloon sized to the diameter of the artery, according to the pre-manipulation trans-operative ultrasound measurement of the artery.

The choice of carotid arteries was based on previous studies evaluating the intimal reaction to BMS and polymer-coated BMS^28^^–^ 30. Studies by Grudtner et al.^14^ have shown that porcine carotid arteries are a suitable model both for evaluating drug-eluting stents in the peripheral bed and for being an alternative technique to implantation in coronary arteries, making it possible to perform selective catheterization, angioplasty, and stent implantation.

Tepe et al.^30^ demonstrated that the implantation of drug-eluting stents in the carotid artery of pigs is a viable and effective technique, but they found high rates of thrombosis, which were correlated with inadequate platelet inhibition after implantation. In our study, the use of AAS 100 mg and clopidogrel 75 mg daily before phase I, together with the absence of thrombotic events, suggests protection from such acute and subacute thrombotic events. In similar animal studies, divergent results were obtained in relation to treatment with or without antiplatelet^14^^,^ 31.

Although the study had some limitations, these were not enough to invalidate the experimental research. The limitations include the use of healthy animals instead of models with stenotic lesions or pre-existing occlusions, the absence of pre-dilation of the carotid arteries with a balloon catheter, and the use of balloon-expandable stents instead of self-expanding stents. The latter, therefore, has greater flexibility and less radial force and is a less potent inducer of local vascular response. However, these limitations did not compromise the overall validity of the results.

One of the limitations of this study was the fact that it was carried out on healthy animals receiving a non-atherogenic diet^14^. At the same time, as observed by França et al.^22^ and Narayanaswamy et al.^17^, intimal hyperplasia in pigs develops after stent implantation, regardless of whether their diet is atherogenic^32^.

The extrapolation of the results to humans is restricted, although it is known in the literature that pigs are similar to humans in terms of lipid profile and vascular response to injury, even though in this study there were no stenotic lesions or pre-existing occlusions associated with the presence of atherosclerotic plaques^32^.

Studies by Schwartz et al.^19^ have shown that it is possible to cause significant damage to the endothelium after balloon angioplasty, just as research by Raudales et al.^20^ confirmed lesions associated with balloon expansion and hyperexpansion during stent implantation, sufficient to cause endothelial damage leading to intimal hyperplasia between seven and 28 days^14^^,^ 15.

Another limitation of this study was that the carotid arteries were not pre-dilated with a balloon catheter, a practice closer to clinical practice, but there was dilation at the time of stent implantation, which could be an additional factor in injury and intimal hyperplasia. However, this method is justified because the study assessed the vascular response induced by the presence of the stent, excluding responses caused by severe trauma secondary to angioplasty^14^^,^ 17 ^,^ 22.

This is one of the few experimental studies to observe the response of balloon-expandable chrome-cobalt stents, with and without polymer coating, developed in Brazil, in porcine carotid arteries using SEM^29^.

Future longitudinal studies could contribute to understanding these results, using self-expanding stents in central and peripheral arteries, such as the femoral arteries for a period longer than 28 days, since self-expanding stents are preferably used for the treatment of atherosclerotic carotid artery disease and peripheral arterial disease^33^^,^ 34. Furthermore, long-term experimental studies are needed to assess whether the effects of biopolymers are beneficial and durable, compared to polymeric devices currently available on the world market^35^.

## Conclusion

The preclinical experimental study in pigs with stents containing PU and PU+RAPA showed complete endothelial coverage by endoluminal neointima of the stent struts, which did not differ from BMS at 28 days. SEM is a method that can be used to evaluate the endoluminal coverage of stent struts. The decision to choose the diameter of the stents based solely on selective angiographic imaging of the carotid artery may be insufficient due to underdsizing and inadequate stent accommodation. Ballooning the spasms may be necessary and effective in maintaining positive remodeling of the artery.

## Data Availability

All data sets were generated or analyzed in the current study.
